# Supplementary material for: Hands Ahead in Mind and Motion: Active Inference in Peripersonal Hand Space
Source: Vision (Basel). 2019 Apr 18;3(2):15. doi: 10.3390/vision3020015 (PMC6802774; doi:10.3390/vision3020015)
Supplement: Supplementary file 1 [file vision-03-00015-s001.zip › vision-465171-s/Supplementary2.docx]

S2: Supplementary Data Analysis

1. Overview

The main analysis presented in the manuscript focus on anticipatory crossmodal congruency effects (aCCE) in verbal RTs. Besides verbal RTs, we collected movement onset and movement times, as well as error rates. Movement onset time refers to the duration between target onset and the hand leaving the starting position. Movement time refers to the duration between movement onset and the first contact with the bottle. Error rates allow for investigation if the verbal RT pattern is affected by a speed accuracy trade-off, while the movement onset and movement times allow one to check if the movement execution was influenced by the response to the tactile stimulation.

2. Experiment 1

Movement onset times and movement times from the 16 participants were analyzed with 2 (orientation) × 2 (distractor) × 2 (stimulation) × 4 (SOA) repeated measure ANOVAs. Only correct trials were included in the analysis. All reported post-hoc comparisons were submitted to a Holm-Bonferroni correction. The analyses were carried out with R (R Core Team, 2016) and the *ez* package [1]. In the case of violations of the assumption of sphericity, *p*-values were submitted to a Greenhouse-Geisser adjustment. Error rates were analyzed using generalized mixed models, using the *lme4* package [2].

2.1. Movement Onset Times

The analysis of movement onset times revealed a main effect for SOA (*F*(1,15) = 15.84, *p* < .001, η_p_² = .51), as well as a significant interaction between SOA and stimulation (*F*(3,45) = 3.71, *p* = .018, η_p_² = .20, all remaining *p*’s ≥ .147). Movement onset times differed significantly for all SOAs (all p’s ≤ .046), however, the difference between SOA2 and SOA3 was not significant (*Mdn*_SOA0_ = 633 ms; *Mdn*_SOA1_ = 702 ms; *Mdn*_SOA2_ = 678 ms; *Mdn*_SOA3_ = 684 ms). Fastest movement onsets were observed at SOA0, that is, when the stimulation was applied before the target was shown. Regarding the interaction between SOA and distractor, post-hoc t-tests showed no differences for stimulation at thumb or index finger for later SOAs. For SOA0, stimulation of the index finger yielded the fastest movement onset times (*Mdn*_SOA0Index_ = 620 ms), however, the differences with stimulations of the index finger at SOA2 (*Mdn*_SOA2Index_ = 673 ms) and with stimulations of the thumb at SOA0 (*Mdn*_SOA0IThumb_ = 646 ms) were not significant. Furthermore, movement onset time after index finger stimulation at SOA1 (*Mdn*_SOA1Index_ = 707 ms) was significantly slower than movement onset time after index finger stimulation at SOA2 (*Mdn*_SOA2Index_ = 673 ms), thumb stimulation at SOA0 (*Mdn*_SOA0IThumb_ = 646 ms), and thumb stimulation at SOA3 (*Mdn*_SOA3IThumb_ = 678 ms).

2.2. Movement Times

The analysis of movement times yielded significant main effects for SOA (*F*(3,45) = 4.56, *p* = .021, η_p_² = .23) and orientation (*F*(1,16) = 9.27, *p* = .008, η_p_² = .38; all remaining p’s ≥ .056). Movement time was significantly faster in the case of the earliest stimulation at SOA0 (*Mdn*_SOA0_ = 1804 ms) compared to all other SOA conditions (*Mdn*_SOA1_ = 1869 ms; *Mdn*_SOA2_ = 1870 ms; *Mdn*_SOA3_ = 1927 ms), however, the differences were not significant after adjusting for multiple tests. Furthermore, movement times were faster when the bottles were presented upright (*Mdn* = 1767 ms) compared to bottles presented upside-down (*Mdn* = 1968 ms).

2.3. Error Rates

The main aim of the error analysis was to investigate whether an aCCE is visible in the error distribution or not, and to check if faster responses in congruent trials were more error-prone. Both error (i.e., participants named the wrong finger) and correct trials of all participants, except the trials without response (59 out of 3072 trials), were coded as 0 (error) or 1 (correct) and entered into a generalized mixed model analysis with a binomial distribution. To simplify the model selection, the combination of the factors stimulation and distractor was recoded into a congruency factor depending on bottle orientation (see Figure 3).

We compared models of increasing complexity with likelihood ratio tests to determine whether the factors SOA, orientation, and congruency were required to account for the error pattern. We kept the error structure simple, applying only a random intercept per participant. After the identification of the null model, we added fixed effects for the experimental factors to the model as long as the likelihood ratio test between the simpler and the more complex model yielded significant results (with α = .05). We only compared nested models differing with respect to one factor. Models with a single fixed effect were compared with the null model, models with two fixed effects were compared with models with one fixed effect, and so on.

The Logit models had the following form:

| $\mathrm{Logit}\left( P \right)=\beta_{0}+\sum_{i=1}^{m} \beta_{i}x_{i}$ | (S1) |
| --- | --- |

Here, $\beta_{0}$ refers to the intercept, $\beta_{i}$ is a weighting parameter for the predictor $x_{i}$. The predictors are the factor levels (e.g., congruent vs. incongruent), while the weights quantify their respective impact on the prediction. Logit estimates are hard to interpret, but they can be transformed to odds ratios using the following relation:

| $\mathrm{OR}=e^{\beta}$ | (S2) |
| --- | --- |

Here, odds ratios can be interpreted as changes in the risk to respond with the wrong (i.e., non stimulated) finger.

The best fitting model involved fixed effects for all factors, as well as an interaction between orientation and congruency (see Table S1). Errors were 20 times more likely in cases of bottles presented upside down compared to upright bottles. Regarding the SOA, most errors occurred at SOA0; at SOA1, errors were 2.28 times less likely. While the main effect for congruency did not reach significance, there was a significant interaction between congruency and orientation. When bottles were presented upright, errors were 12 times more likely in cases of incongruent stimulations. For bottles presented upside down, the risk for an error increased only by a factor of 1.5 in cases of incongruent stimulation. Observed and predicted error rates for the interaction are shown in Table S2.

2.4. Summary

Results regarding verbal RTs (see the results section in the main manuscript) and movement time correspond well with our earlier findings regarding aCCE during pantomimic grasping tasks (see Belardinelli et al., 2018). Regarding the movement onset times, there is a notable difference compared to our earlier results. In contrast to the pantomimic interaction, movement onset times do not decrease linearly with SOA. Longest movement onset times were observed for SOA1, that is, if the stimulation occurred 250 ms after target onset. The analysis of the error rates implies an aCCE as well, however, compared to the effect of bottle orientation, the effects of the assumed aCCE were less pronounced.

**Table S1**. Effect estimates for the best fitting binomial generalized mixed model regarding the error rate from Experiment 1 (df = 8, logLik = -301.6, BIC = 667.4). For easier interpretation, the logit estimates have been transformed to odds. Z statistics for the Wald test and according p-values are presented in the last two columns. Significant effects (α = .05) are indicated with an asterisk. The interaction between congruency and orientation was significant. Incongruent stimulation in cases of bottles presented upright yielded 12 times more errors than congruent stimulation. For bottles presented upside down, the respective risk of an error only increased by 1.5 in cases of incongruent stimulation.

| **Effect** | **Var.** | **Odds** | **95% CI** | **Z** | **p** |
| --- | --- | --- | --- | --- | --- |
| **Random Effects** |  |  |  |  |  |
| Participant | 1.892 |  |  |  |  |
| **Fixed Effects** |  |  |  |  |  |
| Intercept |  | 61.95 | [23.77, 161.44] | 8.445 | < .001* |
| Orientation |  | 20.69 | [2.95, 144.91] | 3.051 | .002 * |
| Congruency |  | 0.68 | [0.41, 1.15] | -1.433 | .151 |
| SOA1 |  | 2.28 | [1.05, 4.96] | 2.094 | .036 * |
| SOA2 |  | 1.35 | [0.70, 2.58] | 0.904 | .365 |
| SOA3 |  | 0.71 | [0.40, 1.25] | -1.172 | .241 |
| Orientation × Congruency |  | 0.11 | [0.01, 0.92] | -2.032 | .042* |

**Table S2**. Observed and predicted error rates for the orientation × congruency interaction in Experiment 1.

| **Effect** | **ER_observed_** | **ER_predicted_** |
| --- | --- | --- |
| **upright bottle** |  |  |
| congruent | 0.002 | 0.002 |
| incongruent | 0.018 | 0.018 |
| **rotated bottle** |  |  |
| congruent | 0.031 | 0.030 |
| incongruent | 0.044 | 0.043 |

3. Experiment 2

We analyzed movement onset and movement times from 21 participants with repeated measure ANOVAs according to the 2 (mapping) × 2 (orientation) × 2 (distractor) × 2 (stimulation) × 3 (SOA) factorial design. Error rates were again analyzed with generalized mixed models.

3.1. Movement Onset Times

Analysis of movement onset times revealed no significant effects (all *p*’s > .076). Movement onset times for the different SOAs (*Mdn*_SOA1_ = 688 ms; *Mdn*_SOA2_ = 679 ms; *Mdn*_SOA3_ = 681 ms) were similar to those from the first experiment.

3.2. Movement Times

Analysis of the movement times revealed significant main effects for mapping (*F*(1,20) = 4.62, *p* = .044, η_p_² = .19), orientation (*F*(1,20) = 29.01, *p* < .001, η_p_² = .59), stimulation (*F*(1,20) = 6.62, *p* = .018, η_p_² = .25), and SOA (*F*(2,40) = 12.63, *p* = .001, η_p_² = .39, all remaining *p*’s ≥ .060). Participants moved faster in the case of the consistent sensorimotor mapping compared to the variable one (*Mdn* = 1621 ms vs. 1733 ms), when bottles were presented upright compared to bottles presented upside-down (*Mdn* = 1636 ms vs. 1718 ms), and in the cases of tactile stimulations of the thumb compared to stimulations of the index finger (*Mdn* = 1662 ms vs. 1692 ms). As in the first experiment, movement times were increased for later SOAs (*Mdn*_SOA1_ = 1628 ms; *Mdn*_SOA2_ = 1660 ms; *Mdn*_SOA3_ = 1742 ms) and all post-hoc comparisons were significant (all *p*’s < .044).

3.3. Error Rates

As in Experiment 1, the error analysis aimed to investigate whether an aCCE is visible within the error distribution and to control for possible speed accuracy trade-offs. Again, both error and correct trials of all participants, except the trials without response (62 out of 6048 trials), were coded as 0 (error) or 1 (correct) and entered into a generalized mixed model analysis with a binomial distribution. The model selection procedure was the same as for Experiment 1, with the only difference being the consideration of the additional mapping factor.

The best fitting model involved only a fixed effect for congruency (see Table S3). Errors were 2.26 times more likely in case of incongruent trials, compared to congruent trials. Again, the predicted error rates fit the observations very well (see Table S4).

**Table S3**. Effect estimates for the best fitting binomial generalized mixed model regarding the error rate from Experiment 2 (df = 3, logLik = -304.3, BIC = 634.7). Logit estimates have been transformed to odds. Z statistics for the Wald test and according p-values are presented in the last two columns. Significant effects (α = .05) are indicated with an asterisk. Only the main effect for congruency was significant, and errors are 2.26 times more likely in incongruent trials.

| **Effect** | **Var.** | **Odds** | **95% CI** | **Z** | **p** |
| --- | --- | --- | --- | --- | --- |
| **Random Effects** |  |  |  |  |  |
| Participant | 0.547 |  |  |  |  |
| **Fixed Effects** |  |  |  |  |  |
| Intercept |  | 225.58 | [122.11, 416.77] | 17.303 | < .001* |
| Congruency |  | 0.44 | [0.25, 0.77] | -2.863 | .004* |

**Table S4**. Observed and predicted error rates for the congruency effect in Experiment 2.

| **Effect** | **ER_observed_** | **ER_predicted_** |
| --- | --- | --- |
| congruent | 0.005 | 0.005 |
| incongruent | 0.013 | 0.012 |

3.4. Summary

The results regarding movement onset and movement times fit well with the results from Experiment 1. However, without SOA0, the significant main effect of SOA on movement onset time vanishes, as well as the interaction between tactile stimulation and SOA. With respect to the error rates, the resulting model for Experiment 2 involves much less effects, which is partially due to the overall reduced error rate in Experiment 2 (1.9% vs. 4.3%) and the according lower variance in the error distribution. Error rates from both experiments do not imply a speed accuracy trade-off, rather it seems that incongruent stimulation increases the error risk.

References

1. Lawrence, M. A. (2015) *ez: easy analysis and visualization of factorial experiments*. https://CRAN.R-project.org/package=ez, R package version 4.3.
2. Bates, D., Maechler, M., Bolker, B. & Walker, S. (2015). Fitting Linear Mixed-Effects Models Using lme4. *J Stat Softw,* 67, 1-48. doi: 10.18637/jss.v067.i01.

| 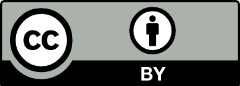 | © 2019 by the authors. Submitted for possible open access publication under the terms and conditions of the Creative Commons Attribution (CC BY) license (http://creativecommons.org/licenses/by/4.0/). |
| --- | --- |
